# Imaging and Image Transfer in Emergency Medicine

**DOI:** 10.1155/2013/952672

**Published:** 2013-12-19

**Authors:** Tobias Lindner, Hein Lamprecht, Efthyvoulos Kyriacou, Raoul Breitkreutz, Stefan Puig, Aristomenis K. Exadaktylos

**Affiliations:** ^1^Department of Emergency Medicine, Campus Virchow, Charité-Universitätsmedizin Berlin, 13353 Berlin, Germany; ^2^Division of Emergency Medicine, Stellenbosch University, Cape Town 7925, South Africa; ^3^Department of Computer Science and Engineering, Frederick University, 3080 Lemesos, Cyprus; ^4^Department of Emergency Medicine, Klinikum Frankfurt Höchst GmbH, 65929 Frankfurt am Main, Germany; ^5^Department of Diagnostic Radiology, Inselspital Bern, University of Bern, Diagnostische, Interventionelle und Pädiatrische Radiologie, 3010 Bern, Switzerland; ^6^Department of Emergency Medicine, Inselspital Bern, University of Bern, 3010 Bern, Switzerland

This special issue presents an international forum for researchers to summarise the most recent developments and ideas in the field of emergency imaging and image transfer, with a special emphasis on the technical and observational results obtained within the last five years.

First of all, we would like to thank all the authors for their contributions. For reasons of space, we were forced to reject other submitted papers. Secondly, we are very much obliged to our reviewers for their excellent and punctual work.

It is a basic duty of emergency medicine to rule out severe illness or to recognise it and treat it at once. Like point-of-care biomarkers, bedside imaging techniques may play a substantial role in coping with this task. In times of increasing pressure on emergency physicians to deliver faster and better results, rational use of imaging techniques during the very first minutes and hours from the time of admission is of the greatest interest. This not only improves outcome and decreases morbidity and mortality but also saves money and enhances patient satisfaction. C. A. Pfortmueller et al. address the issue of quality management on emergency wards. By comparing six different methods of assessing patient satisfaction, they concluded that patient-optimized online assessment performed in the hospital can give a very good response rate. Pfortmueller et al. recommend involving an external institute, as this can save time and money.

Ultrasound (US) is a 24/7 bedside and also prehospital point-of-care imaging tool and was the theme of several studies submitted to this special issue. M. J. El Sayed and E. Zaghrini review current and future indications of preclinical US. They point out that US can help prehospital providers to guide management in several settings (e.g., trauma, cardiopulmonary resuscitation). Despite the great prehospital potential for US, they see problems in developing an adequate training concept for nonphysicians, in order to ensure that they gain and maintain sufficient skills. Moreover, further outcome research is needed to show the benefits of preclinical US. R. Breitkreutz et al. tested the influence of the integration of personalised US on in-hospital patient management. They conducted four substudies on image quality, work distribution, and diagnostic and procedural quality of US in critical care medicine. They found out that integration of personalised US during their regular ward rounds significantly reduced average contact time per patient and lowered the patient referral rate to an echo lab from 20% to 2%.

In a retrospective study enrolling almost 20,000 patients in a level 1 trauma centre, A. Y. Sheng et al. show that the introduction in recent decades of focused assessment with sonography in trauma (FAST) has led to an equivalent reduction in the annual rate of abdominal computed tomography (CT) (around 2%), whereas the overall use of CT in emergency departments has risen during this period. The authors conclude that FAST can avoid unnecessary radiation exposure and also reduce costs in a relevant number of trauma patients. Their future aim is to establish a safe “FAST-only” algorithm. In another ultrasound study, K. Layman and colleagues focus on the use of US in the ED in the evaluation of pain or bleeding in the first trimester of pregnancy. On the basis of three typical cases, they review common pitfalls and discuss key principles of US use in this setting.

Four further papers on ultrasound deal with teaching concepts and protocols for the clinical use of ultrasound, even by inexperienced examiners. T. V. Mai et al. investigate the feasibility of a brief cardiac limited US examination protocol (CLUE) performed by three trainees who have no prior US experience at all and who use a pocket-sized ultrasound device (PUD) under wireless audiovisual guidance from an off-site cardiologist. Data were transmitted through the camera of a phone attached to the PUD. The technical quality of the images and diagnostic accuracy (LV systolic dysfunction, LA enlargement, ultrasound lung comets, and elevated CVP) were compared to clinical routine (on-site echo threshold). Referring to their results, authors conclude that, under these circumstances, a novice can perform the CLUE protocol and facilitate off-site/bedside medical decision making. M. Campo dell'Orto et al. describe a self-made phantom training model using a celluloid table tennis ball for ultrasound-guided pericardiocentesis. On the basis of its evaluation by 67 participants in the course of a focused echocardiography training session, they conclude that their phantom is an effective training tool for acute and critical care physicians. C. Cuca et al. (a member of the same group) evaluated a self-developed interactive online training program for the ultrasound of the thorax, trachea, and lung by comparing the success of independent theoretical skill development through their individual e-learning course compared to a day-long training seminar with personal attendance. They found that e-learning almost equalled scores of classroom-based training with respect to the gain and retention of factual knowledge. They therefore considered that this was set to become a vital part of theoretical training in lung ultrasound. The third paper on training concepts for ultrasound skills from R. Breitkreutz et al.'s very active research group describes the development and assessment of a structured clinical examination curriculum for focused thorax, trachea, and lung (THOLUUSE) ultrasound in emergency and critical care medicine. In a prospective educational study, medical students, anaesthesiologists, and trauma surgeons underwent a one-day training course structured according to their concept. Training included lectures in physiology, the sonoanatomy and sonopathology of the thorax, case presentations, and hands-on training, including ultrasound phantoms, puncture models, and healthy models and selected patients. They concluded that their course significantly improves theoretical and practical skills for the diagnosis of acute thoracic lesions, including pleural effusion/haemothorax and pneumothorax.

Another section of this special issue is dedicated to recent technical developments and their potential benefit in medical care. M. Tabbara et al. introduce the Swiss Limmex Emergency Wristwatch, as launched in 2011. By pushing a button on this watch, the wearer can initiate multiple emergency calls and establish mobile communication with a preselected person, institution, or a search and rescue service. Due to the rising number of elderly people who want to live on their own in their private homes, technical developments like this are of increasing importance. Such devices can assure safe storage and automated or easy transmission of relevant personal medical data, supporting fast and reliable medical treatment in an emergency. In their study, the authors present the results of a survey with 620 answers (response rate 46%) of watch wearers with a mean age of 81.8 years. G. Lindner and A. K. Exadaktylos review previous clinical studies on the accuracy of noninvasive haemoglobin measurements using pulse carboxyoximetry devices and discuss their potential usefulness in different clinical settings, and also in prehospital triage. T. Lindner et al. present the clinical results of the recently introduced PneumoScan, a noninvasive bedside device that uses micropower radar technology to detect pneumothorax. The authors consider that this device might be used in the prehospital setting and for triage in disaster medicine, as the scanner is not examiner dependent like ultrasound and is easy to use only after a short tutorial. However further clinical evaluation is necessary. A. K. Zisakis et al. describe several new and interesting devices that are still in the early phase of clinical testing but one day could help in diagnosing brain injury, measuring intracranial pressure, or even reopening occluded brain vessels. These devices are not exclusive for neurosurgeons or neurologists but also for nonspecialists.

S. P. Whiley et al. present the new Johannesburg trauma protocol which implements the Lodox Statscan, a low dose, full-body X-ray scanner. Its use as a triage tool is illustrated by the description of 63 patients scanned on entry to the ED in a single shift at a level 1 trauma centre. The authors draw the conclusion that this method of imaging can be an efficient and accurate method of triage in underresourced situations. U. J. Gupp et al. ask if the radiation dose to severely injured patients in emergency CT scans can be reduced whilst maintaining image quality. They therefore examined the impact of adaptive statistical iterative reconstruction (ASIR) on CT imaging quality, diagnostic interpretability, and radiation dose reduction for a proven CT acquisition protocol for total body trauma. They compared 30% and 40% of iterative reconstruction (IR) modification in the raw data domain of the routine total body protocol with the routine protocol itself. The authors presented only small numbers of patients in preliminary results and regarded their study as a feasibility evaluation of IR algorithms in an emergency setting. They cautiously concluded that the use of IR algorithms is a promising approach to reduce radiation exposure without compromising radiological interpretability, even in an emergency setting where image quality is paramount.

Finally, K. N. O'Laughlin et al. address the topic of risk stratification for brain herniation after lumbar puncture (LP). Firstly, they asked members of an expert panel to define which head CT findings indicate an increased risk of herniation following LP. Secondly, they used clinical data of a cohort of patients who had a head CT for any reason to assess the ability of history and physical examination to predict those findings. They concluded that no patient history and no findings during clinical examination alone or in combination are adequately sensitive to detect head CT abnormalities believed to predict enhanced potential for brain herniation during LP.

